# Supplementary material for: Ex Situ Synthesis and Characterizations of MoS2/WO3 Heterostructures for Efficient Photocatalytic Degradation of RhB
Source: Nanomaterials (Basel). 2022 Aug 28;12(17):2974. doi: 10.3390/nano12172974 (PMC9458188; doi:10.3390/nano12172974)
Supplement: Supplementary file 1 [file nanomaterials-12-02974-s001.zip › nanomaterials-1859661-supplementary.pdf]

nanomaterials

SUPPORTING INFORMATION

Ex Situ Synthesis and Characterizations of MoS_2/WO_3 Heterostructures for Efficient Photocatalytic Degradation of RhB

Wajeehah Shahid [1], Faryal Idrees [2,*], Muhammad Aamir Iqbal [3], Muhammad Umair Tariq [2], Samiah Shahid [4] and Jeong Ryeol Choi [5,*]

1 Department of Physics, University of Lahore, Lahore 54000, Pakistan;
2 Department of Physics, University of the Punjab, Lahore 54590, Pakistan;
3 School of Materials Science and Engineering, Zhejiang University, Hangzhou 310027, China;
4 Institute of Molecular Biology and Biotechnology, University of Lahore, Lahore 54000, Pakistan;
5 Department of Nanoengineering, Kyonggi University, Suwon 16227, Korea
* Correspondence: faryal.physics@pu.edu.pk (F.I.); jrchoi@kyonggi.ac.kr (J.R.C.)

EDX Spectra:

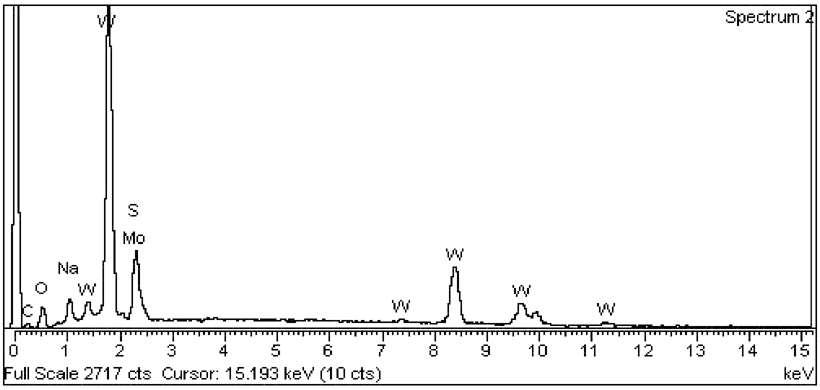

Figure S1. EDX spectra of MoS_2/WO_3 heterostructures.

SEM Analysis:

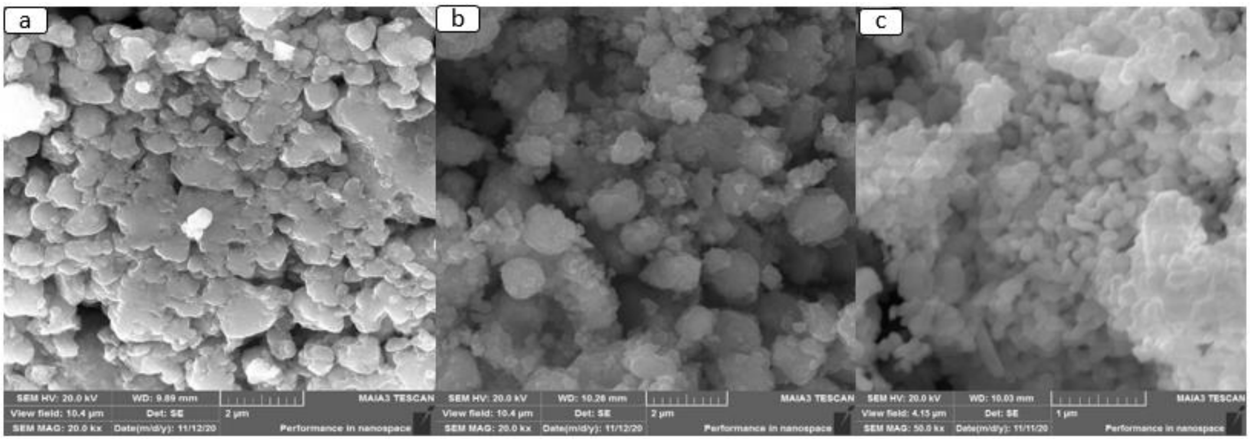

Figure S2. SEM image of (**a**,**b**) MoS_2 and (**c**)WO_3.

Absorption Spectra:

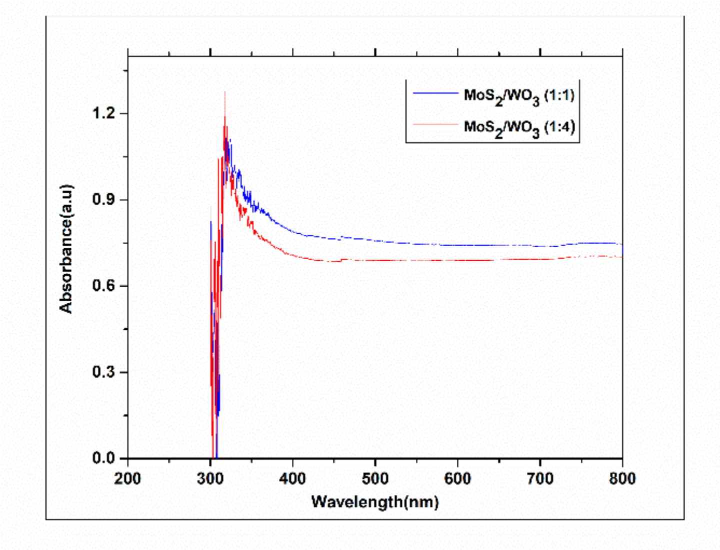

Figure S3. Absorption Spectra of MoS_2/WO_3(1:1) and MoS_2/WO_3(1:4).
